# Treatment of pediatric severe acute myopericarditis with anakinra: a case report and literature review

**DOI:** 10.3389/fped.2025.1544126

**Published:** 2025-04-23

**Authors:** Francesco Licciardi, Federico Fornari, Francesca Ferroni, Carlotta Covizzi, Chiara Riggi, Davide Montin

**Affiliations:** ^1^ Department of Pediatrics and Public Health, Università Degli Studi di Torino, Turin, Italy; ^2^ Immunorheumatology Unit, Ospedale Infantile Regina Margherita, Città Della Salute e Della Scienza, Turin, Italy; ^3^ Postgraduate School of Pediatrics, Università Degli Studi di Torino, Turin, Italy; ^4^ Division of Pediatric Cardiology, Ospedale Infantile Regina Margherita, Città Della Salute e Della Scienza, Turin, Italy

**Keywords:** acute myocarditis, anakinra, case report, myopericarditis, pericarditis, severe inflammation

## Abstract

Acute myocarditis (AM) is an inflammation of the myocardium with a rapid onset of typically <1 month. The use of anakinra (ANK) for treating inflammatory AM in adults has been recently described; however, while some reports are promising, its efficacy remains debated. Here, we present a case of severe AM with concomitant systemic symptoms [fever, elevated C-reactive protein (CRP)] in a pediatric patient who was successfully treated with high-dose ANK. A literature review of similar published cases is also presented. A 14-year-old boy was admitted for AM with concomitant pericarditis. At disease onset, the patient presented with high fever and elevated CRP (163 mg/L) and troponin I (14,816 ng/L). Treatment with ibuprofen (30 mg/kg/day), intravenous immunoglobulin (80 g in 24 h), and colchicine (0.5 mg per day) were initiated without benefit and with further worsening of contractile function [Ejection Fraction (EF) 26%]. Consequently, inotropic support and intravenous methylprednisolone were started, leading to a partial improvement of EF (45%). Due to the inability to reduce inotropic support, a rescue treatment with ANK (7 mg/kg/day) in continuous intravenous infusion was started, resulting in progressive improvement and normalization of left ventricular systolic function. Our literature review identified five case reports of pediatric AM successfully treated with ANK. Most cases presented elevated inflammatory markers (ferritin and CRP) and/or concomitant pericarditis. We conclude that ANK, especially at high doses, may be useful for treating severe pediatric AM, particularly when associated with severe inflammation and/or pericarditis.

## 1 Introduction

Acute myocarditis (AM) is an inflammation of the myocardium with an onset of typically <1 month caused by abnormal immunoreactivity, drug or toxic substance exposure, or infection (1). Its clinical spectrum is variable, ranging from minor illness to high-risk cardiac conditions, including severe heart failure, refractory arrhythmias, cardiogenic shock, and sudden cardiac death (2, 3). AM treatment is mostly supportive, and evidence supporting the role of immunosuppression as a treatment strategy is limited (4).

The past ten years have seen a growing body of evidence, both in murine models (5) and in humans (6), showing an activation of the NOD-like receptor protein 3 (NLRP3) inflammasome during AM, ultimately leading to increased interleukin (IL)-1 levels in affected patients. These observations provided the rationale for using IL-1 inhibitors, especially anakinra (ANK), for treating AM. ANK is a recombinant human IL-1 receptor antagonist that neutralizes the biological activity of IL-1α and IL-1β by competitive binding to the IL-1 receptor type I (IL-1RI) (7, 8).

The use of ANK in treating inflammatory AM has primarily been described in adults; however, while some reports are promising, its efficacy remains debated (9). In the pediatric setting, ANK has primarily been used to treat Multisystem Inflammatory Syndrome in Children (MIS-C), a severe autoinflammatory condition following SARS-CoV-2 infection in children. MIS-C is characterized by persistent fever, mucocutaneous signs resembling Kawasaki disease, and heart failure (10). Published reports on ANK in pediatric patients with AM but without MIS-C are sparse.

Here, we report the case of a 14-year-old patient with severe AM and pericarditis who responded to IL-1 inhibition with ANK. We also discuss this case in light of a literature review of currently available evidence on the use of ANK in children with AM.

## 2 Case report

A 14-year-old boy (weight 57 kg) with sudden onset of thoracic and interscapular pain and fever was admitted to the local emergency room. The patient was previously healthy, played sports at a competitive level, and had no family history of heart disease.

**Figure 1:**
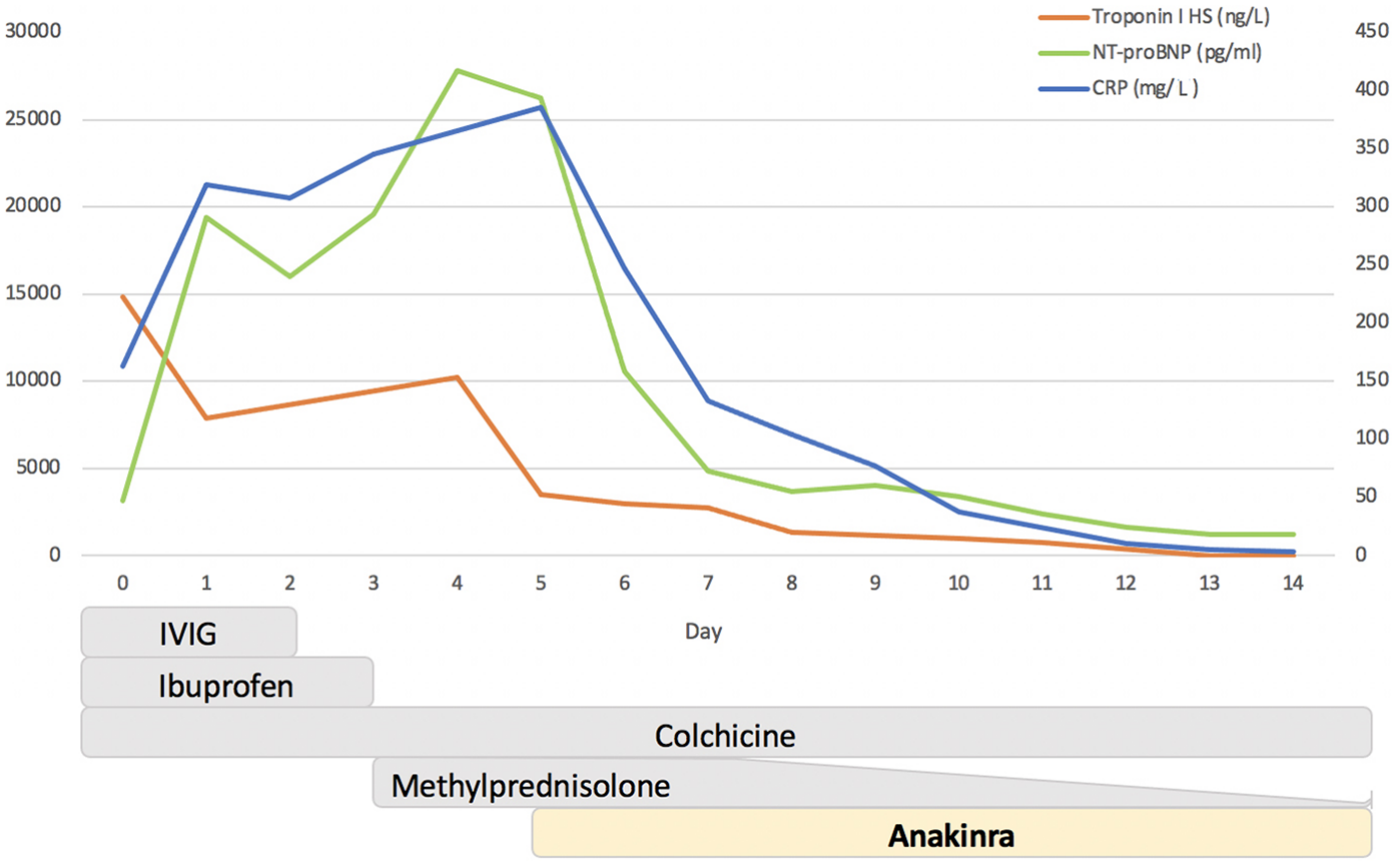
Timeline of changes in C-reactive protein (CRP), troponin I, and NT-proBNP after therapeutic interventions. The *Y*-axis on the left refers to troponin I (ng/L) and NT-proBNP (pg/ml), while the *Y*-axis on the right refers to CRP (mg/L). IVIG, intravenous immunoglobulin.

Vital signs on admission were essentially normal, and physical examination found a hemodynamically stable patient with rhythmic cardiac activity and no pathological heart murmurs. Electrocardiogram (ECG) was pathological, showing sinus rhythm with ascending S-T elevation in V4–V6, DI, DII, and AVF. Blood tests showed neutrophil leukocytosis (WBC count 21,160; neutrophils 83%), increased inflammatory response markers [C-reactive protein (CRP) 163 mg/L], and elevation in cardiac enzyme levels (high-sensitivity troponin I 14,816 ng/L); NT-proBNP was 3,140 ng/L (normal <450 ng/L) (Day 0; Figure 1).

Echocardiogram showed hypokinesis of the posterolateral wall and mild-to-moderate impaired global left ventricular contraction [estimated B-mode ejection fraction (EF) 45%–48%]. Mild mitral insufficiency was present. Right ventricular function appeared normal. Mild posterior pericardial effusion of 5–6 mm was present. Blood cultures assessing the presence of pathogens commonly associated with myocarditis (CMV, EBV, Enterovirus, Parvovirus, Adenovirus, HSV1-2, Coxsackie, SARS-CoV-2) were negative. Myocardial biopsy was not performed.

In the setting of myopericarditis of unknown cause, treatment with ibuprofen (30 mg/kg per day), intravenous immunoglobulin (IVIG) (80 g in 24 h), and colchicine (0.5 mg per day) was started. On Day 2, the patient's clinical condition deteriorated with the resumption of fever, hypotension, lung rales, contraction of diuresis, hepatomegaly, and worsening of contractile function (EF 40%). The patient was transferred to the ICU and placed under CPAP; treatment with furosemide and inotropes (adrenaline, milrinone, and levosimendan) was started.

Further worsening of the patient's clinical condition was observed on Day 3, with a severe reduction of contractile function (EF 26%) and signs of low cardiac output (diuresis contraction, diffuse ST elevation on ECG, and increased serum lactate up to 12 mmol/L). Augmentation of inotropic support and intubation were necessary. Immunomodulatory therapy was potentiated with bolus corticosteroid treatment (methylprednisolone 600 mg/day). Ibuprofen was withdrawn, while colchicine was maintained.

On Day 5, the patient's condition was stationary, with echocardiographic improvement in contractile function (EF 45%). However, a clinical picture of heart failure persisted with the inability to wean the patient from inotropic therapy and mechanical ventilation. Blood tests demonstrated the persistence of a systemic inflammatory response (CRP 386 mg/L) and myocardial damage (troponin I 2,710 ng/L); NT-proBNP was 26,265 ng/L (Figure 1).

Despite the hyperinflammatory state (ferritin 667 ng/ml), no clinical or laboratory criteria suggestive of Still's disease or Macrophage Activation Syndrome were present. Considering the inflammatory markers, the persistence of pericardial effusion, and the inability to decrease inotrope support, a rescue treatment with ANK 7 mg/kg/day in continuous intravenous infusion was started on Day 5 (Figure 1).

A progressive improvement on a clinical level was subsequently observed, alongside a reduction in inflammatory markers and normalization of left ventricle systolic function. The patient was extubated after 3 days (Day 8), and inotropic treatment was discontinued after 9 days (Day 14). Treatment with ANK was reduced at 100 mg/day and switched to subcutaneous administration on Day 10 and discontinued on Day 15. Steroid therapy was progressively tapered without relapse (Figure 1).

**Figure 2:**
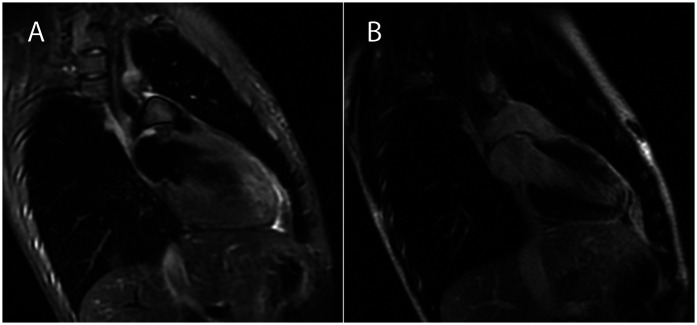
Cardiac magnetic resonance imaging performed 1 month after acute myocarditis onset, two-chamber view: T2 weighted imaging **(A)** enables visualization of myocardial edema in the anterior wall concordant with regional late gadolinium enhancement **(B)** with an epi and mid-myocardial distribution.

Cardiac magnetic resonance imaging (MRI) performed 1 month after onset (Figures 2A,B) showed a non-dilated left ventricle with mildly reduced systolic function [EF 52% [normal range 56%–76%], EDIV 66 ml/m^2^ [normal range 56–104 ml/m^2^], EDSI 31.5 ml/m^2^ [normal range 16–40 ml/m^2^], and SV 57 ml] and no involvement of right ventricle. Edema and late gadolinium enhancement (LGE) were present at the mid-lateral, infero-lateral wall, and mid-apical anterior wall with intramyocardial and subepicardial patterns (see Figures 2A,B). At 40 days after onset, the patient was stable and asymptomatic. CRP, troponin, and NT-proBNP levels had normalized. Sinus rhythm on ECG was observed; negative T waves persisted in DI, aVL, and V4–V6. Echocardiogram showed normalized global and segmental systolic function and no pericardial effusion. The patient was subsequently discharged with ongoing outpatient cardiological and immunological follow-up.

No myocarditis flare was observed during follow-up. After 6 months off therapy, the patient experienced a mild flare of pericarditis but without myocardial involvement. The patient had no fever or signs suggestive of systemic JIA (no rash, no arthritis). Blood tests showed mild elevation of CRP (15 mg/L), with normal ferritin and ESR, and negative autoantibodies. A screening for recent infections (coxasachiae, EBV, adenovirus) excluded a post-infectious pericarditis. ANK 100 mg/day was re-started, leading to rapid improvement. Now, 18 months after re-starting ANK, the patient is in complete remission while receiving a low dose of Anakinra (100 mg two days/week).

## 3 Literature review

### 3.1 Search strategy

Our literature review adhered to the Preferred Reporting Items for Systematic reviews and Meta-Analyses (PRISMA) 2020 guidelines (11). A systematic literature search was conducted in PubMed on August 5th, 2024, without language restriction using predefined MESH terms “AND” and “OR.” The following search terms were used: [“myocardic” (All Fields) OR “myocarditis” (MeSH Terms) OR “myocarditis” (All Fields) OR “myocarditides” (All Fields)] AND [“interleukin 1 receptor antagonist protein” (MeSH Terms) OR “interleukin 1 receptor antagonist protein” (All Fields) OR “ANK” (All Fields)].

### 3.2 Eligibility criteria

Studies were included if they fulfilled the following criteria: patients (age <18 years) with a confirmed diagnosis of AM via cardiac MRI or myocardial biopsy; use of ANK to treat myocarditis during the acute phase. Case reports, case series, and prospective and retrospective studies were included. Studies were excluded if they reported on MIS-C or other COVID-19-related cardiovascular complications, involved animal testing, or were review articles.

### 3.3 Data extraction

Titles and abstracts were initially assessed for inclusion by two reviewers (Fr Li and Fe Fo), with discrepancies arbitrated by the senior author (Fr Li). Full-text screening of relevant articles was conducted independently by Fr Li and Fe Fo to decide which satisfied the inclusion criteria.

Data extracted from the selected studies and combined in a shared Excel spreadsheet included demographic data (age and gender), study design, publication year, patient comorbidities, symptoms, length of hospitalization, duration of management, treatment used, and patient outcomes.

### 3.4 Results

Our search string identified 78 studies, of which five were deemed relevant according to the inclusion criteria. Most were case reports (12–15). Only one of ten patients in the retrospective case series (16) was diagnosed with AM and included here. Patient characteristics, study source, and clinical histories of the five patients plus our patient are summarized in Table 1.

**Table 1:** Published case reports of acute myocarditis treated with anakinra, including this case report.

	Butin et al. 2013 (12)	Luconi et al. 2015 (13)	Meneghel et al. 2020 (14)	Maunier et al. 2022 (16)	Pernaa et al. 2024 (15)	This publication
Article type	Case report	Case report	Case report	Retrospective case series	Case report	Case report
No. pts	1	1	1	1^a^	1	1
Age (years)	6	17	2	1	17	14
Diagnosis	Parvovirus B19 Myocarditis	Still's disease-associated myocarditis	MAS-associated myocarditis	Parvovirus B19 Myocarditis	Recurrent myopericarditis	Acute myopericarditis
CRP zenith	436 mg/L	395 mg/L	100 mg/L	10 mg/L	57 mg/L	386 mg/L
Concomitant HLH/MAS or Still's disease	sHLH	Still's disease	MAS in Still's disease	No	No	No
Troponin peak^b^	n/a	2.5 ng/ml	n/a	n/a	2.1 ng/ml	2.7 ng/ml
Cardiogenic shock	Yes	No	Yes	Yes	No	Yes
Concomitant pericarditis	Yes, with tamponade	Yes	SCLS	n/a	No	Yes
Arrhythmia	No	No	No	Yes	No	No
Supportive treatment	Dobutamine	None	Inotropic support (unspecified), ECMO	Milrinone, amiodarone, levosimedan, ECMO	None	Adrenaline, milrinone, levosimendan
Anakinra dose	2 mg/kg/day titrated to 8 mg/kg/day	100 mg/day	2 mg/kg/day titrated to 8 mg/kg/day	4 mg/kg/day	100 mg/day	7 mg/kg/day
Time from admission to Anakinra start	23 days	A few days after admission (unspecified)	2 days	3 days	n/a	5 days
Concomitant systemic therapy	IVIG, steroids, CSA	Steroids	Steroids, CSA, IVIG	IVIG	No	IVIG, steroids, colchicine
Side effects	Not reported	Not reported	Neutropenia	Not reported	Not reported	Not reported

CSA, cyclosporine; ECMO, extracorporeal membrane oxygenation; IVIG, intravenous immunoglobulin; MAS, Macrophage Activation Syndrome; n/a, not available; SCLS, systemic capillary leak-syndrome; sHLH, secondary hemophagocytic lymphohistiocytosis.

^a^ In Maunier et al., 9/10 patients were excluded because they were affected by MIS-C.

^b^ Troponin peak (normal value <0.03 ng/ml).

Patient ages ranged from 1 to 17 years. Two patients were diagnosed with myopericarditis and four with myocarditis. Four patients required inotropic support, two of whom also required extracorporeal membrane oxygenation (ECMO). ANK dosage was reported as 100 mg/day (2 studies) or between 2 mg/kg/day and 8 mg/kg/day (4 studies). All five patients responded to ANK, and neutropenia was the only adverse event reported (14) (Table 1).

## 4 Discussion

We report the case of a pediatric patient with AM associated with hyperinflammation who responded to IL-1 inhibition with ANK. ANK has been successfully used to treat myocarditis in adults (17–20); however, the recent ARAMIS trial failed to demonstrate its efficacy in a cohort of adult patients with AM (9). In the ARAMIS trial, 120 patients with myocarditis diagnosed by MRI and elevated cardiac enzymes were randomized to receive either 100 mg/day of ANK or a placebo. Overall, the incidence of severe adverse events was low in both groups and was equally distributed. However, the study lacked sufficient statistical power to detect differences in a cohort of patients who generally had very mild myocarditis without systemic symptoms; only 10% of the enrolled patients had an EF of <50%. Furthermore, the dose of ANK administered, at <2 mg/kg/day, was low (9).

In the pediatric population, the use of ANK in myocarditis is reported far less frequently, with only 5 cases published to date. Therefore, this is the sixth case report of ANK in a pediatric patient with AM. Notably, most patients had severe myocarditis, with 67% requiring inotropic support and 33% needing ECMO. Despite their overall severity, all patients responded to ANK, with significant improvements in both systemic symptoms and cardiac function (12–16).

Our patient experienced a rapid onset of severe myocarditis, accompanied by significantly elevated inflammatory markers. Notably, three of the previously published cases treated with ANK also had myocarditis in the context of concomitant hemophagocytic lymphohistiocytosis/Macrophage Activation Syndrome (HLH/MAS) or Still's disease (12–14). Myocarditis is rare at the presentation of Still disease and MAS with an estimated incidence <5% in both diseases (14, 21, 22). The efficacy of ANK in treating MAS has been demonstrated in various studies and is commonly used in everyday practice (23). Interestingly, our patient did not meet the criteria for MAS but exhibited only a persistent high fever with high RCP. This suggests that ANK may be beneficial not only for myocarditis secondary to systemic illnesses such as MAS and Still's disease but also for AM with concomitant prominent systemic inflammation. According to published studies, only 27% of patients with AM have significantly elevated inflammatory markers, such as CRP (24). ANK is likely beneficial in cases of AM associated with systemic inflammation because it acts at multiple levels. First, like its role in MAS, ANK antagonizes IL-1, a key cytokine involved in the inflammatory process (7). Additionally, ANK modulates blood pressure and improves overall circulatory performance (25). Secondly, ANK may exert a direct effect on the myocardium, counteracting the increase of IL-1 due to persistent inflammasome activation in cardiomyocytes (6, 7).

Our patient also exhibited moderately elevated ferritin levels, indicative of a systemic hyperinflammatory state. This finding is observed in other cases included in our review, and it is reasonable to infer that increased levels of ferritin, along with other inflammatory markers, confirm the presence of a hyperinflammatory condition. This supports the rationale for using IL-1 antagonists in this context (6).

Another interesting factor observed in our case and two previously reported cases is the concomitant presence of pericarditis (13, 15). While data in pediatrics are scarce, in adults, the estimated incidence of elevated troponin in patients with pericarditis is 28.8%; nevertheless, most of these patients have normal left ventricular function. The incidence of cardiac insufficiency secondary to myocarditis in patients with pericarditis is around 5% (peri-myocarditis). Patients with peri-myocarditis have a good long-term prognosis if they survive the acute phase of the disease. Recently, one adult patient with peri-myocarditis was successfully treated with ANK, confirming that it may be a promising therapeutic option even for older patients (26, 27). The efficacy of ANK in treating idiopathic pericarditis has been confirmed in numerous retrospective case series (28, 29). In our case, ANK may have had a positive effect on pericardial effusion, ultimately improving cardiac contraction. Given its proven effects on systemic inflammation and its role in treating pericarditis, it is possible that ANK could be particularly effective in a subgroup of patients with AM associated with pericarditis and/or systemic inflammation.

In this report, ANK was administered at a high dose (i.e., 7 mg/kg/day) via intravenous infusion. It is worth noting that in two previously published cases, the treating physicians initially started with a lower dose (2 mg/kg/day) but quickly increased it due to cardiac deterioration (12, 14). These observations suggest that ANK could be initiated at a high dose in cases of AM and that a lack of response to a lower dose does not necessarily indicate treatment failure. Interestingly, data suggest a higher dose of ANK may be more effective in treating cardiac dysfunction in MIS-C, especially in severe cases (30).

Neither our patient nor the previously published cases of AM treated with ANK experienced severe adverse events, with only one case of transient neutropenia reported (14). Of note, according to previous experience with ANK in MIS-C patients, the drug seems safe even at high doses, with increased ALT being the most frequent side effect, which usually disappears after dose adjustment (30).

This study has some limitations. First, in this case, as in others, ANK was administered after IVIG and steroids. Second, there is significant heterogeneity in how treatment response was assessed in previous reports, making it impossible to quantify the drug response in more detail.

## 5 Conclusion

In summary, we report the case of a patient experiencing life-threatening AM associated with hyperinflammation, who showed significant cardiological and systemic improvement following treatment with high-dose ANK. A systematic literature review highlighted five other pediatric cases of AM treated with ANK. The results appear promising in this limited series, particularly in patients with AM associated with severe inflammation and/or pericarditis. Further studies are needed to evaluate the safety and efficacy of IL-1 inhibitors in this context and to define the optimal dose and timing of ANK treatment in pediatric myocarditis.

## Data Availability

The original contributions presented in the study are included in the article/Supplementary Material, further inquiries can be directed to the corresponding author.
